# Biogenic Silver Nanoparticles Mediated by Phytoconstituents of the Edible Spice *Pimpinella anisum*: Characterization and In Vitro, In Vivo, and In Silico Biological Evaluation

**DOI:** 10.1111/1750-3841.71388

**Published:** 2026-08-31

**Authors:** Ibtissam Laib, Anis Ben Ali, Soundes Guedda, Nardjes Guedda, Naglaa A. Abd‐Elkarim, Yousef Benaissa, Nariman Essmat, Elhafnaoui Lanez, Mohamed F. Abouelenein, Ahmed I. Osman, Alaa Mahfouz, Ahmed K. Rashwan

**Affiliations:** ^1^ Department of Cellular and Molecular Biology El Oued University El Oued Algeria; ^2^ Laboratory of Biodiversity and Biotechnology Applications in Agriculture University of El Oued El Oued Algeria; ^3^ Laboratory of Biology, Environment and Health, Faculty of Natural and Life Sciences University of El Oued El Oued Algeria; ^4^ Department of Food and Dairy Sciences, Faculty of Agriculture Qena University Qena Egypt; ^5^ VPRS Laboratory, Chemistry Department, Faculty of Mathematics and Matter Sciences University of KASDI Merbah Ouargla Algeria; ^6^ Department of Pharmacology and Toxicology, Faculty of Pharmacy Zagazig University Zagazig Egypt; ^7^ Faculty of Exact Sciences, Department of Chemistry, VTRS Laboratory University of El Oued El Oued Algeria; ^8^ Department of Insurance and Risk Management, College of Business Imam Mohammad Ibn Saud Islamic University (IMSIU) Riyadh, Riyadh Saudi Arabia; ^9^ School of Sciences, Psychology, Arts and Humanities, Computing, Engineering, and Sport Canterbury Christ Church University Canterbury UK; ^10^ Medicinal and Aromatic Plants Department Desert Research Center Cairo Egypt; ^11^ Department of Food Science and Nutrition, College of Biosystems Engineering and Food Science Zhejiang University Hangzhou China

**Keywords:** analgesic, antidepressant, anti‐inflammatory, antioxidant, molecular docking, multi‐target therapy, *Pimpinella anisum*, silver nanoparticles

## Abstract

This study evaluated the aqueous extract of *Pimpinella anisum* seeds (AE‐PAS) and its green‐synthesized silver nanoparticles (PA‐AgNPs) using integrated in vitro, in vivo, and in silico approaches. LC–MS analysis identified 26 phytoconstituents, mainly sinapic acid (15.78%), hispidulin (15.35%), caffeic acid (11.09%), and rutin (8.86%). Total phenolic and flavonoid contents were 45.2 ± 2.1 mg GAE/g DE and 28.7 ± 1.5 mg QE/g DE, respectively. UV–Vis spectroscopy, scanning electron microscopy (SEM), Fourier‐transform infrared (FTIR) spectroscopy, and X‐ray diffraction (XRD) supported nanoparticle formation, indicating dry‐state particle or agglomerate features of approximately 60–90 nm, a surface plasmon resonance (SPR) peak at 421 nm, 50.81% crystallinity, and a crystallite‐domain size of 22.57 ± 1.2 nm. AE‐PAS showed measurable antioxidant and albumin‐denaturation‐inhibitory responses (DPPH IC_50_ = 130.63 ± 1.3 µg/mL; albumin‐denaturation IC_50_ = 721.79 ± 1.7 µg/mL). PA‐AgNPs produced measurable inhibition zones in agar well diffusion screening, but antibacterial potency could not be established because MIC and MBC were not determined. The PA‐AgNP DPPH result lacked a nanoparticle‐only blank and should be regarded as an apparent assay‐specific estimate. Hemolysis was concentration dependent, increasing from 5.20% at 50 µg/mL to 29.93% at 100 µg/mL. In behavioral models, PA‐AgNPs (0.1 mg/kg) produced measurable antidepressant‐like and analgesic responses; these findings represent preliminary behavioral evidence rather than confirmed efficacy. Molecular docking predicted favorable binding scores (−8.0 to −9.6 kcal/mol) for selected extract phytochemicals against MAO‐A/B, COX‐1/2, and TNF‐α but did not establish target inhibition or a biological mechanism. Overall, the findings are preliminary and require comprehensive physicochemical characterization, appropriate silver‐ion and material controls, quantitative antimicrobial testing, pharmacokinetic and mechanistic validation, and expanded safety assessment before biomedical translation.

## 1 Introduction

Silver nanoparticles (AgNPs) have been investigated in medicine, biotechnology, and pharmaceutical research because their surface, catalytic, optical, and electrical properties can be modified through particle composition and synthesis conditions. Numerous studies have reported antioxidant, antimicrobial, anti‐inflammatory, analgesic, and neurobiological responses associated with AgNP formulations; however, the magnitude and safety of these responses depend strongly on particle size, surface chemistry, aggregation, silver‐ion release, dose, and the experimental model used (Zhang et al. 2024; Naganthran et al. 2022; Barabadi et al. 2023). Conventional physical and chemical fabrication methods may require substantial energy or hazardous reagents, which has encouraged the development of plant‐mediated synthesis approaches (Tarannum et al. 2019; Dhaka et al. 2023). In these approaches, plant constituents such as flavonoids, phenolic acids, proteins, and polysaccharides may participate in silver‐ion reduction and surface association (Verma et al. 2023; Hanna et al. 2025; Kirubakaran et al. 2026). Nevertheless, green synthesis alone does not establish biocompatibility, stability, or therapeutic performance. Comprehensive physicochemical characterization, suitable controls, quantitative biological assays, and cautious mechanistic interpretation remain necessary. Molecular docking can generate hypotheses regarding possible interactions between free phytochemicals and protein targets, but docking scores do not establish target inhibition, an in vivo mechanism, or the behavior of an intact nanoparticle formulation (Pinzi and Rastelli 2019).

*Pimpinella anisum* L. (anise), an aromatic seed from the Apiaceae family, is used for culinary and traditional medicinal purposes. Its seeds contain phenolic acids, flavonoids, and other phytochemicals that have been associated in previous studies with antioxidant, anti‐inflammatory, antimicrobial, neurobiological, and digestive responses (Sun et al. 2019; Jawed et al. 2025). Previous investigations have mainly examined crude extracts and essential oils, whereas the use of anise‐derived constituents in plant‐mediated AgNP synthesis remains comparatively less explored. Incorporating plant constituents into a nanoparticle formulation may alter their dispersion, surface interactions, and biological behavior; however, improvements in bioavailability, safety, or therapeutic efficacy cannot be assumed without direct pharmacokinetic, toxicological, and mechanistic evidence. Accordingly, anise‐mediated AgNPs should be evaluated as an experimental nanobiosystem rather than presumed to be safe or therapeutically superior (Ghamarsoorat et al. 2025; Zayed et al. 2020).

Despite growing interest in green‐synthesized AgNPs, relatively few studies have combined phytochemical profiling, basic nanoparticle characterization, in vitro screening, animal behavioral testing, and molecular docking for *P. anisum*‐mediated materials. The present study, therefore, characterized the aqueous extract of *P. anisum* seeds (AE‐PAS) by LC–MS and evaluated PA‐AgNP formation using UV–Vis, Fourier transform infrared (FTIR), x‐ray diffraction (XRD), and scanning electron microscope (SEM). Antioxidant, albumin‐denaturation, antibacterial, photoprotective, and hemolytic responses were screened in vitro, followed by behavioral assessment of antidepressant‐like and analgesic responses in rats. Molecular docking was used to explore predicted interactions of selected extract phytochemicals with MAO‐A/B, COX‐1/2, and TNF‐α. This integrated design permits a preliminary comparison of phytochemical composition, nanoparticle‐associated features, and measured biological responses. It does not establish a molecular mechanism, clinical efficacy, improved bioavailability, or biomedical safety, and the findings must be interpreted considering the characterization and control limitations described below.

## 2 Materials and Methods

### 2.1 Plant Materials, Chemicals, and Reagents

Anise (*P. anisum* L.) seeds were collected in November 2024 from El Eulma (36°09′54″ N, 5°41′25″ E), Sétif Province, Algeria, and taxonomically verified by Professor Atef Chouikh (University of El Oued). Seeds were washed, dried, ground, and stored for further use. All chemicals were analytical grade from Sigma‐Aldrich (St. Louis, MO, USA), including silver nitrate (AgNO_3_, 99.9%) for nanoparticle synthesis, sulpiride, paracetamol, indomethacin, various acids and buffers, DPPH, Folin–Ciocalteu (FC) reagent, aluminum chloride, ascorbic acid, methanol, sodium dodecyl sulfate (SDS), ferric chloride, gallic acid, quercetin, and other reagents, all used in corresponding assays to ensure experimental accuracy and reproducibility.

### 2.2 Experimental Animals and Housing Conditions

Thirty healthy adult male albino rats (160–250 g) were obtained from the Pasteur Institute of Algiers and housed at the Department of Molecular and Cellular Biology, University of El Oued, Algeria. Rats were acclimated for 1 month under standardized conditions (19°C ± 1°C, 64% ± 2% humidity, 12 h light/dark cycle) with ad libitum access to standard chow and water (five per cage). All procedures followed ethical guidelines approved by the University of El Oued Ethics Committee (approval no. 04/S.C./FL/NS/EU/2025). Only male rats were used in the present study; therefore, the findings may not generalize to female animals or reveal possible sex‐dependent behavioral or safety responses.

### 2.3 Preparation of the Aqueous Extract

AE‐PAS was prepared according to the method described in a previously published study (Pontes et al. 2019), with minor modifications. The method involved suspending 10 g of dried seed powder from *P. anisum* in 100 mL of distilled water for a period of 24 h at room temperature (RT) using maceration. After 24 h, the resulting solution was filtered using a Whatman filter, and the resulting liquid (i.e., filtrate) was evaporated to dryness under reduced pressure using a rotary evaporator. The final product was weighed, recorded, and stored at 4°C until further use.

### 2.4 LC–MS Analysis of the Aqueous Extract

The components of *P. anisum* water extract were analyzed using UPLC–ESI–MS/MS on a Shimadzu LCMS‐8040 (Kyoto, Japan) with ultrahigh performance mass spectrometry (UFMS). Separation was achieved on a Restek Ultra C18 column (150 × 4.6 mm^2^, 3 µm) at 40°C. The mobile phase comprised A (0.1% formic acid in water) and B (methanol) with a linear gradient: A at 98% (0–0.2 min), decreasing to 25% by 2.5 min, 0% by 4 min, held at 0% until 7 min, then returned to 98% at 7.1 min and equilibrated to 12 min. Flow rate was 0.2 mL/min, injection volume 5 µL. ESI conditions: CID gas 230 kPa, conversion dynode −6 kV, desolvation line 250°C, heat block 400°C, nebulizing gas 3 L/min, drying gas 10 L/min.

### 2.5 Determination of Phenolic and Flavonoid Content

Total phenolic content (TPC) was assessed by the FC colorimetric assay method (khatoon et al. 2013). In summary, 200 µL each of the standard or AE‐PAS was combined with 1000 µL of diluted FC reagent (10×) and 800 µL of Na_2_CO_3_ (3%). The resultant mixture was then vortexed thoroughly and incubated for 30 min at 40°C; after incubation, the absorbance was recorded at 725 nm using a Jenway (UK) UV/Vis 6850 spectrophotometer. TPC was evaluated via a calibration curve of gallic acid standard and expressed as mg GAE/g DE (mg of gallic acid equivalents per gram of dry extract). Measurements were made in triplicate.

The aluminum chloride (AlCl_3_) method of determining total flavonoid content (TFC) was modified from previous work (Kobacy et al. 2025) as follows: 1 mL of quercetin as standard or AE‐PAS was combined with 75 µL of sodium nitrite (NaNO_2_ 5%) and reacted for 6 min at RT; 150 µL of AlCl_3_ (6%) was then added and allowed to incubate for an additional 6 min at RT. Following this, 0.5 mL of sodium hydroxide (NaOH) (4%) was added to the mixture, resulting in a total volume of 3 mL when combined with distilled water. The reaction mixture was incubated for 10 min in the dark at RT before the absorbance was read at 510 nm using a UV/Vis 6850 spectrophotometer (Jenway, UK). TFC is calculated based on a calibration curve of quercetin and is reported as mg QE/g DE (mg of quercetin equivalents per gram of dried extract). All analyses were performed in triplicate.

### 2.6 Green Synthesis of PA‐AgNPs

The synthesis of PA‐AgNPs was carried out through a green synthesis method published by Ghamarsoorat et al. (2025). In this process, 10 mL of AE‐PAS was added gradually to 90 mL of 10 mM AgNO_3_ solution with constant stirring; after several minutes, a brownish color would appear, revealing that nanoparticles had formed. After forming, the reaction mixture was incubated at 60°C in the dark for 24 h, which ensured complete reduction had occurred. The nanoparticles produced were collected via centrifugation followed by successive washes with ethanol and distilled water to remove any residual contaminants; after drying, the final PA‐AgNPs were available for characterization and experimental use.

### 2.7 Characterization of PA‐AgNPs

#### 2.7.1 Morphology Analysis and UV–Vis Spectroscopy

The surface morphology of PA‐AgNPs was examined using a Phenom Pro Desktop SEM (Phenom‐World B.V., Thermo Fisher Scientific, Eindhoven, the Netherlands). The instrument provided an optical magnification range of 20–134×, an electron‐optical magnification range of 160–150,000×, a maximum digital zoom of 12×, and selectable accelerating voltages of 5, 10, and 15 kV. Samples were prepared by mounting small amounts of PA‐AgNPs onto carbon double‐sided adhesive tape affixed to an aluminum stub and subsequently lightly sputter‐coated with gold using an ion sputter coater. Nanoparticle distributions were evaluated from the SEM micrographs with the use of ImageJ software (NIH, Bethesda, MD, USA), and these data were statistically evaluated via OriginPro 2018 (OriginLab Corporation, Northampton, MA, USA). The spectrophotometric properties of PA‐AgNPs were assessed via UV–Vis Spectrophotometry (Shimadzu UV‐1800, Japan) at 200–800 nm (Ghamarsoorat et al. 2025; Naganthran et al. 2022).

#### 2.7.2 ATR‐FTIR Spectroscopy and XRD

FTIR spectroscopy, using an Agilent Cary 630 Spectrometer (Agilent Technologies), was used to determine the functional group composition and bonding characteristics of PA‐AgNPs. The sample was placed directly on the ATR plate, and uniform pressure was applied to promote good contact. Spectra were collected as the average of eight scans at a resolution of 16 cm^−1^ and over the spectral range of 400–4000 cm^−1^ (Fayed et al. 2024). Characterization of the crystallinity of PA‐AgNPs was performed using the PROTO AXRD Benchtop diffractometer with Cu*K*α radiation (*λ* = 0.154281 nm), operated at 30 kV/20 mA, with a step size of 0.05° (2*θ*) over an angular range of 20°–80° (2*θ*). The mean crystallite size (*D*) of PA‐AgNPs was calculated using the Scherrer formula, *D* = (*Kλ*)/(*β*cos*θ*); where *K* is the shape factor (0.9), *λ* is the x‐ray wavelength (0.154281 nm = Cu*K*α), *β* is the full width at half maximum (FWHM), *θ* is the Bragg angle (Metwaly et al. 2025). The degree of crystallinity (%) was calculated as the ratio of the area of the integrated crystalline peaks (*A*_crystalline_) and the total area of the diffractogram (*A*_total_) after baseline correction:

$$\mathrm{Degree}\;\mathrm{of}\mathrm{crystallinity}\left(\%\right)=\frac{A_{\mathrm{crystalline}}}{A_{\mathrm{total}}}\;\times 100$$

### 2.8 In Vitro Biological Activities of PA‐AgNPs

#### 2.8.1 Antioxidant Activity

The in vitro antioxidant activity of AE‐PAS and PA‐AgNPs was assessed using three assays: DPPH radical scavenging, reducing power activity (RPA), and phosphomolybdate (TMAC) (khatoon et al. 2013), all performed in triplicate. For the DPPH assay, 1 mL of DPPH• solution was mixed with 1 mL of AE‐PAS or PA‐AgNPs, with ascorbic acid as a positive control. After vortexing, samples were incubated for 30 min at RT in the dark, and absorbance was measured at 517 nm. Nanoparticle‐only blanks were not included for the PA‐AgNPs samples.

The RPA involved reacting samples at various concentrations with 2.5 mL of 0.2 M phosphate buffer (pH 6.6) and 2.5 mL of 1% potassium ferricyanide at 37°C for 20 min. After adding 2.5 mL of 10% trichloroacetic acid and centrifuging at 3000 rpm for 10 min, 2.5 mL of the supernatant was mixed with 2.5 mL of distilled water and 0.5 mL of 0.1% FeCl_3_, and absorbance was read at 700 nm. Total antioxidant capacity (TAC) was measured using the phosphomolybdate assay, where 0.3 mL of sample was combined with 3 mL of reagent (0.6 M H_2_SO_4_, 28 mM sodium phosphate, 4 mM ammonium molybdate), heated at 95°C for 90 min, cooled, and read at 695 nm. Results were expressed as mg AAE/g DE (milligram ascorbic acid equivalents per gram dry extract).

#### 2.8.2 Photoprotective Activity

The photoprotective potential of AE‐PAS and PA‐AgNPs was assessed by spectrophotometric determination of sun protection factor (SPF), following the method described in a previous study (Laib et al. 2024). Samples were prepared at 1 mg/mL in ethanol for uniform dispersion, and absorbance was measured from 290 to 320 nm at 5 nm intervals. A commercial sunscreen (Avène) was used as a positive control. SPF values were calculated using the following equation (Malsawmtluangi et al. 2013):

$$\mathrm{SPF}\;\mathrm{spectrophotometric}=\mathrm{CF}\times \sum_{290}^{320}\times \mathrm{EE}\left(\lambda \right)\times I\left(\lambda \right)\times \mathrm{Abs}\left(\lambda \right)$$

where CF = 10, EE(*λ*) is the erythemogenic effect, *I*(*λ*) the solar intensity, and Abs(*λ*) the absorbance. Measurements were performed in triplicate.

#### 2.8.3 Anti‐Inflammatory Activity

The in vitro anti‐inflammatory activity of AE‐PAS and PA‐AgNPs was evaluated using the egg albumin denaturation method (Naveed et al. 2022). Each 5 mL reaction mixture contained 200 µL fresh hen's egg albumin, 2.8 mL phosphate buffer (pH 6.4), and 2 mL of the test sample (100–500 µg/mL). Distilled water served as a negative control, and Aspegic at the same concentrations was used as a reference. Mixtures were incubated at 37°C for 15 min, followed by heat‐induced denaturation at 70°C for 5 min. After cooling to RT, absorbance was measured at 660 nm using a UV–Vis spectrophotometer, with the vehicle as blank. The inhibition percentage of albumin denaturation was calculated using the standard formula:

$$\%\;\mathrm{Inhibition}=\frac{A_{\mathrm{control}}-A_{\mathrm{sample}}}{A_{\mathrm{conrol}}}\;\times 100$$

where *A*_control_ refers to the absorbance of the control solution (distilled water), whereas *A*_sample_ corresponds to the absorbance of the AE‐PAS, PA‐AgNPs, or Aspegic.

#### 2.8.4 Hemolytic Activity

The hemolytic activities of AE‐PAS and PA‐AgNPs were assessed following the method described in a previous study (Laib et al. 2024). Blood (5 mL) was collected in EDTA tubes, centrifuged at 1000 rpm for 10 min at 4°C, and the washed erythrocytes were stored at 4°C for 6 h. Erythrocyte suspensions were prepared by serial dilution, and 100 µL of either AE‐PAS or PA‐AgNPs at concentrations of 50, 75, and 100 µg/mL was added to the erythrocyte suspensions. PBS (100 µL) served as the negative control, while SDS at the corresponding concentrations served as the positive control. Samples were incubated at 37°C for 60 min, diluted to 1 mL with PBS, centrifuged at 3000 rpm for 3–5 min, and supernatant absorbance was measured at 540 nm. Hemolysis (%) was calculated as

$$\mathrm{Hemolysis}\;\left(\%\right)=\frac{A_{\mathrm{control}}-A_{\mathrm{sample}}}{A_{\mathrm{conrol}}}\;\times 100$$

#### 2.8.5 Antibacterial Activity

The antibacterial activity of AE‐PAS and PA‐AgNPs was tested against *Pseudomonas aeruginosa* (ATCC 27853), *Staphylococcus aureus* (ATCC 25923), *Bacillus subtilis* (ATCC 25973), *Klebsiella pneumoniae* (ATCC 13885), and *Escherichia coli* (ATCC 25922), obtained from the Pasteur Institute of Algiers, Algeria. Using the agar well diffusion method (Kobacy et al. 2025), bacterial suspensions (10^6^ CFU/mL) were spread on Mueller–Hinton agar plates. Wells (6 mm) were filled with 10 µL of test samples in 5% DMSO at various concentrations. Amoxicillin (10 µg/mL) and cephalexin (30 µg/mL) were positive controls, and 5% DMSO was the negative control. Plates were incubated for 24 h at 37°C, and inhibition zones were measured.

### 2.9 In Vivo Biological Activities of AE‐PAS and PA‐AgNPs

#### 2.9.1 Experimental Design, Dose Selection, and Animal Grouping

The AE‐PAS doses of 40 and 80 mg/kg were selected as exploratory doses below the 100 and 200 mg/kg oral doses previously evaluated for *P. anisum* extract in mice (Es‐safi et al. 2021). The PA‐AgNP doses of 0.1 and 0.2 mg/kg were selected as exploratory low doses below the 0.5–10 mg/kg range used in a repeated‐dose rat study of green‐synthesized AgNPs (Tareq et al. 2022). Because the cited studies used different species, formulations, administration routes, and exposure schedules, they provide contextual rather than direct dose justification. The present study therefore does not establish that the selected doses were safe or optimal. The substantially lower mass doses used for PA‐AgNPs should not be interpreted as evidence of enhanced bioavailability, cellular uptake, potency, or safety, because these properties were not directly measured. All treatments were administered intraperitoneally (i.p.) in a volume of 2.5 mL/kg body weight using 0.9% sterile saline as the vehicle. Thirty healthy adult male albino rats were randomly allocated to six groups (*n* = 5 per group) using a computer‐generated randomization sequence. Behavioral assessments were conducted by two observers blinded to treatment allocation. Animals showing illness or abnormal baseline behavior before testing were to be excluded; no animals met the exclusion criteria in the present study.

Sulpiride (10 mg/kg, i.p.) was included as a pharmacological comparator according to the original experimental design. Its use should not be interpreted as representing the conventional antidepressant positive control for the forced swim test (FST) or tail suspension test (TST), or as evidence that AE‐PAS or PA‐AgNPs act through dopamine D2/D3 receptors. Its inclusion provided a reference response for the FST and TST. The use of sulpiride should not be interpreted as evidence that AE‐PAS or PA‐AgNPs act through dopamine D2/D3 receptors, MAO inhibition, or any other monoaminergic mechanism, because receptor activity, MAO activity, and neurotransmitter concentrations were not measured in the present study.

##### 2.9.1.1 Forced Swim Test

The FST was conducted as an adapted rat procedure based on established rat FST methodology, with a pretest session 24 h before the 6‐min test session (Slattery and Cryan 2012). The pretest duration and behavioral‐scoring schedule used in the present study were protocol modifications. Briefly, FST consists of a cylindrical container with dimensions of 20 cm in diameter, 30 cm in height, and filled with 15 cm of water at 26°C ± 1°C. To enhance test sensitivity, a 10‐min pretest session was conducted 24 h before the main experiment to establish baseline behavioral despair. On the testing day, male albino rats received the following treatments: AE‐PAS (40 or 80 mg/kg), PA‐AgNPs (0.1 or 0.2 mg/kg), sulpiride (10 mg/kg, positive control), or 0.9% saline (negative control). All drugs were administered intraperitoneally (i.p.) in a volume of 2.5 mL/kg of 0.9% saline. All treatments were injected 30 min before exposure to the FST, then the animals were placed in the water‐filled cylinder and observed for 6 min. The first minute served as an acclimatization period, after which mobility time was recorded. Substances with antidepressant‐like activity were expected to increase mobility time and, correspondingly, reduce immobility time relative to the saline‐treated group.

##### 2.9.1.2 Tail Suspension Test

An adapted tail‐suspension procedure was used to quantify mobility‐ and immobility‐related behavior in rats. The test apparatus consisted of an enclosed chamber with white acrylic walls measuring 20 × 40 × 60 cm^3^, with one side left open to permit video recording and behavioral observation. Before testing, rats received AE‐PAS at 40 or 80 mg/kg, PA‐AgNPs at 0.1 or 0.2 mg/kg, sulpiride at 10 mg/kg as a pharmacological comparator, or 0.9% saline as the negative control. All treatments were administered intraperitoneally at an injection volume of 2.5 mL/kg, 60 min before behavioral assessment. For testing, each rat was individually suspended by the distal portion of the tail using a secure specialized support that prevented the animal from reaching nearby surfaces. Each session lasted 6 min and was video‐recorded for subsequent behavioral assessment. The first minute was considered an acclimatization period and was excluded from the quantitative analysis. Mobility time was recorded during the remaining 5 min. Mobility was defined as active body movements, including attempts to escape, swinging of the body, movement of the forelimbs or hindlimbs, and torsional movements of the trunk. Immobility was defined as the absence of active escape‐directed movements, with the animal remaining passively suspended and displaying only movements required for respiration. Behavioral scoring was conducted by observers blinded to the treatment allocation. Increased mobility and a corresponding reduction in immobility were interpreted as an antidepressant‐like behavioral response under the conditions of this exploratory procedure. However, because the conventional tail‐suspension test was developed and primarily validated in mice, its application in rats in the present study should be regarded as an adapted exploratory behavioral measure rather than a fully validated rat antidepressant assay (Berrocoso et al. 2013; Can et al. 2012).

#### 2.9.2 Analgesic Activity

##### 2.9.2.1 Hot Plate Test

The hot plate test, a standard method for evaluating pain response in rodents exposed to a heated surface, was employed to assess the analgesic activity of the tested compounds (Ben Ali et al. 2024). Male albino rats received the following treatments: AE‐PAS (40 or 80 mg/kg) and PA‐AgNPs (0.1 or 0.2 mg/kg), paracetamol (10 mg/kg, as positive control), or 0.9% saline (as negative control). Drugs were injected intraperitoneally (i.p.) in 2.5 mL/kg 0.9% saline. Thirty minutes later, each rat was placed on a hot plate maintained at 55°C. The latency time (in seconds) was measured from the moment of placement on the plate until the appearance of a pain response, such as hind paw licking or jumping. To prevent thermal injury, a cutoff time of 30 sec was established.

##### 2.9.2.2 Acetic Acid–Induced Writhing Test

The acetic acid–induced writhing test was employed to evaluate peripheral analgesic activity. Pain response was quantified by counting the total number of abdominal constrictions (writhes) occurring over a 30‐min observation period, commencing 5 min after injection of acetic acid. Before acetic acid administration, animals were treated with AE‐PAS (40 or 80 mg/kg) and PA‐AgNPs (0.1 or 0.2 mg/kg), indomethacin (10 mg/kg, as reference standard), or 0.9% saline (as negative control). Drugs were injected intraperitoneally (i.p.) in 2.5 mL/kg 0.9% saline (Chouikh et al. 2024). The percentage inhibition of writhing (IW%) was estimated according to the following equation:

$$\mathrm{IW}\;\left(\%\right)=\frac{M_{\mathrm{control}}-M_{\mathrm{sample}}}{M_{\mathrm{conrol}}}\;\times 100$$

where *M*_control_ is the mean number of writhes in the control group, and *M*_sample_ is the mean number of writhes in the treated group (AE‐PAS or PA‐AgNPs).

### 2.10 Molecular Docking Study of Bioactive Compounds of *P. anisum*

To explore possible ligand–target interactions relevant to the behavioral and in vitro findings, molecular docking simulations were performed for nine phytochemicals identified by LC–MS: hispidulin, sinapic acid, caffeic acid, ferulic acid, catechin, apigenin, luteolin, naringenin, and resveratrol. Structures were obtained from PubChem and prepared in PDBQT format using AutoDock Tools, with polar hydrogens added and Gasteiger charges assigned (Mehda et al. 2025). Protein structures were retrieved from the Protein Data Bank: MAO‐A (2Z5X), MAO‐B (1GOS), COX‐1 (3KK6), COX‐2 (4PH9), and TNF‐α (9OJS). Proteins were preprocessed by removing water molecules and selected heteroatoms, after which hydrogens and charges were added. Docking grids were defined to encompass the reported binding sites, with parameters provided in Table S1. The docking analysis was performed on free phytochemicals, not on nanoparticle‐bound complexes.

Docking was performed with AutoDock Vina (v1.1.2), using 50 runs for each ligand–protein pair. Predicted binding scores were reported in kcal/mol. Re‐docking of native ligands produced RMSD values below 2 Å (Table S2), supporting internal consistency of the docking protocol but not validating biological activity or target inhibition. Hydrogen‐bond and hydrophobic‐contact patterns were examined using Discovery Studio Visualizer (v21.1). The docking results were used only to generate hypotheses concerning possible phytochemical–protein interactions. Attempts to perform molecular‐dynamics simulations for the nanoparticle formulations produced insufficient convergence and inconsistent trajectories under the tested parameters; consequently, those results are not reported or used for interpretation.

### 2.11 Statistical Analysis

All data were analyzed using SPSS Statistics software (version 23.0; IBM Corp., Armonk, NY, USA), and image processing with quantitative analysis was performed using ImageJ software (NIH, Bethesda, MD, USA). Results are expressed as mean ± standard deviation (SD). Intergroup comparisons were carried out by one‐way analysis of variance (ANOVA) followed by Tukey's post hoc test, with statistical significance accepted at *p* < 0.05.

## 3 Results and Discussion

### 3.1 Phytochemical Profiling of the AE‐PAS by LC–MS

LC–MS‐based relative quantification of AE‐PAS identified 26 phytoconstituents, including phenolic acids, flavonoids, glycosides, and terpenoids. The predominant detected compounds were sinapic acid (15.78%), hispidulin (15.35%), caffeic acid (11.09%), rutin (8.86%), catechin (8.47%), ferulic acid (8.37%), and myricetin‐3‐rhamnoside (7.21%), which together represented 75.13% of the relative concentrations reported in Table 1 (Table 1 and Figure S1). This composition is consistent with the measured TPC (45.2 ± 2.1 mg GAE/g DE) and TFC (28.7 ± 1.5 mg QE/g DE). Phenolic acids and flavonoids can participate in silver‐ion reduction and surface association during plant‐mediated nanoparticle synthesis, as reported previously (Ghamarsoorat et al. 2025; Hu et al. 2025; Farooqi et al. 2024). However, the contribution of each individual compound to nanoparticle formation, capping, stability, or biological activity was not isolated in the present study. Antioxidant, anti‐inflammatory, antibacterial, and behavioral results are therefore discussed in their respective sections without assigning the measured responses to specific compounds or molecular pathways. Previous literature provides broader context for the antioxidant behavior of phenolic compounds and for possible inflammation‐ and nociception‐related activities of phenolic acids; these reports do not establish that the same mechanisms occurred in the present study (Vuolo et al. 2019; Theodosis‐Nobelos et al. 2023).

**TABLE 1:** Phytoconstituents of *Pimpinella anisum* seed aqueous extract identified by LC–MS.

ID#	Name	Formula	ESI charge	CE (V)	Ret. time (min)	Area	Height	Relative conc. (%)
1	Catechin (−)	C_15_H_14_O_6_	+	−13	8.012	660,597	84,251	8.47
2	Chrysin‐6‐C‐glucoside	C_21_H_20_O_9_	+	−25	7.830	87,644	17,318	1.12
3	Myricetin	C_15_H_10_O_8_	+	−20	7.030	35,403	8016	0.45
4	Myricetin‐3‐rhamnose	C_27_H_30_O_17_	+	−44	6.985	561,820	52,863	7.21
5	Oroxylin A	C_16_H_12_O_5_	+	−22	6.892	101,955	15,022	1.31
6	Quercetin‐3‐arabinose	C_20_H_18_O_11_	+	−15	6.920	8490	1109	0.11
7	Quercetin‐3‐glucoside	C_21_H_20_O_12_	+	−18	6.824	118,810	21,690	1.52
8	Tiliroside	C_30_H_26_O_13_	+	−18	6.865	32,976	5572	0.42
9	Apigenin	C_15_H_10_O_5_	+	−33	7.287	10,954	2272	0.14
10	Hispidulin	C_16_H_12_O_6_	+	−10	6.952	1,196,353	141,917	15.35
11	Chrysin	C_15_H_10_O_4_	+	−27	7.344	55,470	12,425	0.71
12	Epicatechin	C_15_H_14_O_6_	+	−15	7.091	23,619	4437	0.30
13	Ferulic acid	C_10_H_10_O_4_	+	−16	7.035	652,507	74,525	8.37
14	Luteolin	C_15_H_10_O_6_	+	−32	7.099	3318	1362	0.04
15	Oleanolic acid	C_30_H_48_O_3_	+	−15	6.913	34,094	2304	0.44
16	Oleuropein	C_25_H_32_O_13_	+	−15	7.866	22,929	4319	0.29
17	Quercetin	C_15_H_10_O_7_	+	−52	6.825	10,698	2911	0.14
18	Resveratrol	C_14_H_12_O_3_	+	−22	6.885	144,241	17,629	1.85
19	Rutin	C_27_H_30_O_16_	+	−10	6.806	690,625	137,563	8.86
20	Sinapic acid	C_11_H_12_O_5_	+	−23	6.898	1,229,812	172,003	15.78
21	Naringenin	C_15_H_12_O_5_	+	−43	6.333	5634	1708	0.07
22	Thymol	C_10_H_14_O	+	−18	7.169	39,469	2244	0.51
23	Vanillin	C_8_H_8_O_3_	−	−15	6.849	32,917	6698	0.42
24	Caffeic acid	C_9_H_8_O_4_	−	34	6.742	864,210	227,826	11.09
25	*p*‐Coumaric acid	C_9_H_8_O_3_	−	28	6.879	112,126	18,064	1.44
26	Salicylic acid	C_7_H_6_O_3_	−	37	7.104	193,615	20,444	2.48

### 3.2 Morphology, Size, and UV–Vis Spectroscopy Analysis

**FIGURE 1:**
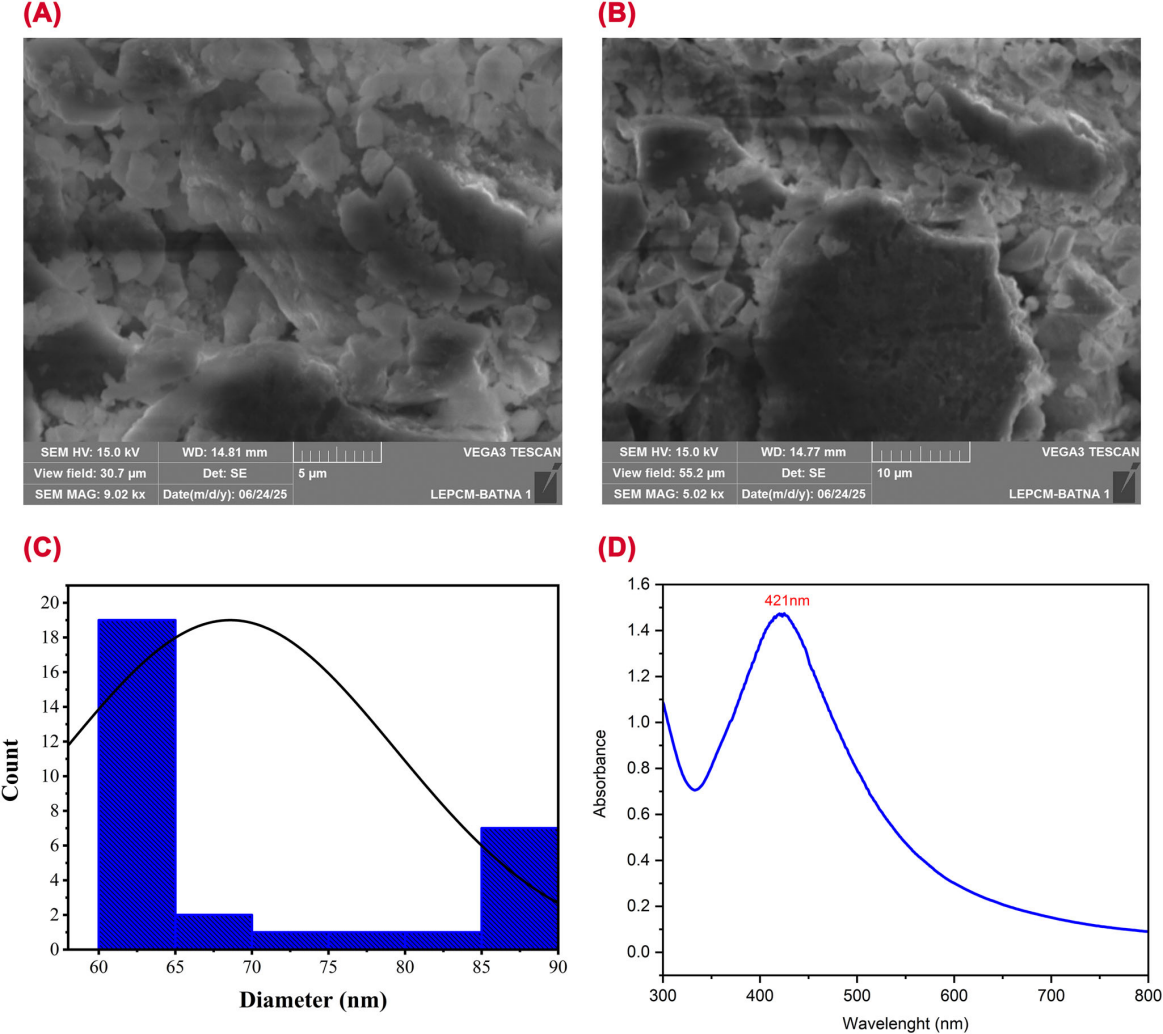
(A) Scanning electron microscopy (SEM) image of silver nanoparticles‐based aqueous extract of *Pimpinella anisum* L. seed (PA‐AgNPs) at 9.02k× magnification (Scale bar: 5 µm), (**B**) SEM image of PA‐AgNPs at 5.02k× magnification (scale bar: 10 µm), (**C**) particle size distribution histogram of PA‐AgNPs were calculated with the scale using the image analysis ImageJ software, and the obtained data were analyzed using OriginPro 2018, and (**D**) UV–Vis absorption spectrum of PA‐AgNPs, showing the characteristic surface plasmon resonance (SPR) peak at 421 nm.

UV–Vis spectroscopy and SEM provided evidence supporting the formation and dry‐state morphology of PA‐AgNP‐containing material (Figure 1A–D). SEM images showed bright nanoscale features associated with a dried organic matrix derived from AE‐PAS. ImageJ measurements from the available SEM micrographs indicated apparent dry‐state particle or agglomerate dimensions mainly between approximately 60 and 90 nm, with an estimated central range near 65–70 nm. ImageJ measurements from the SEM micrographs describe apparent dry‐state particles or agglomerate dimensions. Because DLS and PDI analyses were not performed, these measurements cannot be interpreted as hydrodynamic diameter, a formal polydispersity index, or evidence of colloidal monodispersity (Bhattacharjee 2016). The observed dry‐state morphology is broadly consistent with plant‐mediated silver‐containing nanomaterials, although such comparisons cannot replace direct colloidal characterization (Verma et al. 2023; Metwaly et al. 2025). Similar morphology and dry‐state heterogeneity have been discussed for plant‐mediated silver‐containing materials, but such comparisons cannot substitute for DLS, PDI, zeta‐potential, TEM, or stability measurements (Fayed et al. 2024; Tarannum et al. 2019).

UV–Vis spectroscopy showed a broad absorption maximum at 421 nm (Figure 1D), consistent with a surface‐plasmon‐resonance band reported for silver‐containing nanoparticles produced by plant‐mediated synthesis (Ghamarsoorat et al. 2025; AlSalhi et al. 2016; Dhaka et al. 2023). The band supports nanoparticle formation but does not, by itself, establish particle concentration, monodispersity, hydrodynamic diameter, or colloidal stability. Band broadening may be influenced by particle‐size heterogeneity, aggregation, particle shape, and the surrounding matrix. Phytochemicals present in AE‐PAS may have participated in silver‐ion reduction and surface association; however, the specific compounds and binding modes responsible for these processes were not directly determined (Fayed et al. 2024; Wang et al. 2022). Previous studies have likewise noted that surface plasmon resonance (SPR)‐band broadening can accompany particle‐size heterogeneity or aggregation, although optical spectra alone cannot quantify these properties (Chandak and Nagime 2025; Naveed et al. 2022).

### 3.3 XRD and FTIR Spectroscopy

**FIGURE 2:**
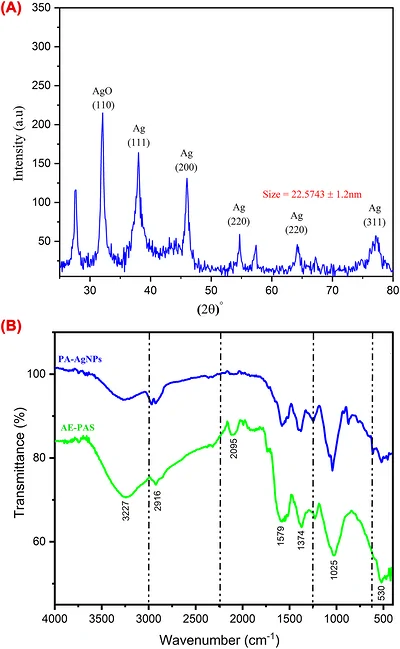
(**A**) X‐ray diffraction (XRD) pattern of silver nanoparticles‐based aqueous extract of *Pimpinella anisum* L. seed (PA‐AgNPs), and (**B**) Fourier‐transform infrared (FTIR) spectra of the pure aqueous extract of *P. anisum* L. seed and PA‐AgNPs. AE‐PAS, aqueous extract of *P. anisum* seeds.

XRD was used to examine the crystalline features of the synthesized material (Figure 2A). The diffractogram contained reflections assigned in the original analysis to face‐centered‐cubic metallic silver and a silver‐oxide‐associated contribution near 32° 2*θ*, supporting the description of the material as an Ag/silver‐oxide‐containing nanocomposite (Ghamarsoorat et al. 2025; AlSalhi et al. 2016; Dhaka et al. 2023; Wang et al. 2022). The mean crystallite‐domain size calculated using the Debye–Scherrer equation was 22.57 ± 1.2 nm. This XRD‐derived domain size is smaller than the 60–90 nm apparent dry‐state features measured from SEM, which may reflect multicrystalline particles, aggregation during drying, and associated organic material. SEM does not provide hydrodynamic diameter, and the difference between XRD and SEM values should not be interpreted as evidence of a specific capping‐layer thickness. The calculated crystallinity was 50.81%, indicating contributions from both crystalline and non‐crystalline components under the applied analysis. The crystallite‐domain size does not by itself demonstrate quantum confinement or superior biological performance. Complementary TEM and more detailed phase analysis would be required for definitive particle and phase assignment (Metwaly et al. 2025; Naganthran et al. 2022; Azzi et al. 2024). Reports on silver/silver‐oxide nanomaterials provide context for the possible phase contribution, but the biological implications of that phase were not tested here (Barabadi et al. 2023).

ATR‐FTIR spectra of AE‐PAS and PA‐AgNPs are shown in Figure 2B. AE‐PAS displayed bands near 3227, 2916, 1579, 1374, and 1025 cm^−1^, consistent with O–H, aliphatic C–H, carbonyl/aromatic, and C–O‐containing functional groups reported for plant extracts (Fayed et al. 2024; AlSalhi et al. 2016). Changes in band position or intensity after synthesis are consistent with participation of extract constituents in silver‐ion reduction or surface association but do not identify individual capping compounds or prove a specific binding mechanism. A low‐frequency band near 530 cm^−1^ may be compatible with an Ag–O vibration and is broadly consistent with the silver‐oxide‐associated XRD contribution (Ravichandran et al. 2016). The spectra therefore support the presence of an organic‐associated silver‐containing material; however, electrostatic surface charge, steric stabilization, and long‐term colloidal stability cannot be established from FTIR alone and require zeta‐potential, DLS/PDI, release, and stability measurements. Plant‐derived hydroxyl, carbonyl, and related functional groups have been reported to participate in silver‐ion reduction and surface association during green AgNP synthesis. However, the individual compounds and binding mechanisms responsible for the present material were not directly resolved (Verma et al. 2023; Flieger et al. 2021).

### 3.4 In Vitro Biological Activities of AE‐PAS and PA‐AgNPs

#### 3.4.1 Antioxidant Activity

**FIGURE 3:**
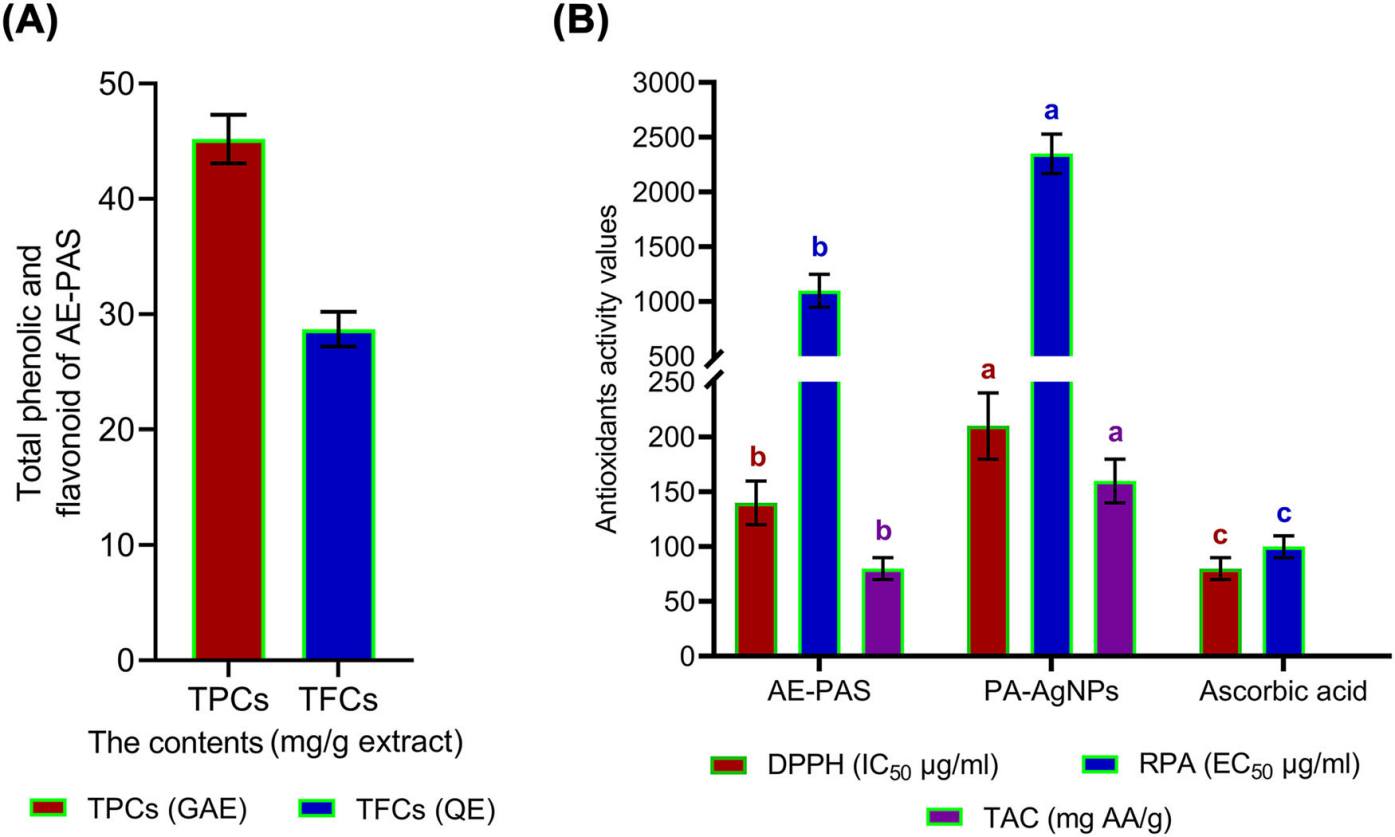
(**A**) Total phenolic content (TPC) and total flavonoid content (TFC) of aqueous extract of *Pimpinella anisum* L. seed (AE‐PAS), (**B**) in vitro antioxidant activity of AE‐PAS and PA‐AgNPs using reducing power activity (RPA), DPPH radical scavenging assay, and total antioxidant capacity (TAC). Values are expressed as mean ± standard deviation (*n* = 3). Different letters (in the same color) indicate significant differences (*p* < 0.05) as determined by one‐way ANOVA followed by post hoc analysis with Tukey's test. µg/mL, microgram/millilitres; AE‐PAS, aqueous extract of *P. anisum* seeds; EC_50_, half maximal effective concentration; GAE, gallic acid equivalents; IC_50_, half maximal inhibitory concentration; mg/g, milligram/gram; QE, quercetin equivalent.

The TPC and TFC of AE‐PAS were quantified as GAE and QE, respectively (Figure 3A). The TPC was found to be 45.2 ± 2.1 mg GAE/g DE, whereas the TFC was 28.7 ± 1.5 mg QE/g DE. These results indicate that AE‐PAS contains substantial phenolic and flavonoid compounds, known for their antioxidant properties. Compared to other plant extracts, these values are typical for aqueous extractions, where water selectively extracts hydrophilic compounds. These findings provide a foundation for further exploration of AE‐PAS's pharmacological potential and its antioxidant effects in biological assays. The antioxidant activity of AE‐PAS and PA‐AgNPs was systematically evaluated using complementary in vitro assays based on different reaction mechanisms and compared with the reference antioxidant ascorbic acid (Figure 3B). This comparative approach was adopted to elucidate the impact of nanoparticle formulation on the redox behavior of plant‐derived phytochemicals. AE‐PAS exhibited measurable antioxidant activity, with a DPPH radical scavenging IC_50_ of 130.63 ± 1.3 µg/mL, a reducing power EC_50_ of 1121.60 ± 1.6 µg/mL, and a TAC of 114.22 ± 2.7 mg AAE/g. These activities are consistent with the LC–MS profile, which revealed a high abundance of phenolic acids (sinapic, caffeic, and ferulic acids) and flavonoids (hispidulin, rutin, catechin, and myricetin derivatives). Phenolic and flavonoid compounds can contribute to antioxidant responses through hydrogen‐atom‐transfer and electron‐transfer reactions, although the contribution of individual AE‐PAS compounds was not determined in the present study (Prior et al. 2005). The FTIR results indicate the presence of functional‐group‐associated bands but do not independently establish the antioxidant reaction mechanism.

For PA‐AgNPs, the uncorrected DPPH assay yielded an apparent IC_50_ of 177.41 ± 1.7 µg/mL, whereas the reducing‐power EC_50_ was 2323.92 ± 1.32 µg/mL and the phosphomolybdate result was 117.93 ± 0.71 mg AAE/g DE. Because a nanoparticle‐only blank was not included, the PA‐AgNP DPPH value may include contributions from light scattering or intrinsic nanoparticle absorbance and should therefore be treated as an apparent, assay‐specific estimate rather than a fully corrected scavenging value. Surface‐associated phytochemicals and particle‐related physicochemical effects may influence reaction accessibility, but enhanced chemical stability, sustained activity, or superior bio‐interface compatibility were not directly measured. The complementary assays provide different redox‐related readouts; however, they do not eliminate potential optical interference in the DPPH measurement. Ascorbic acid showed substantially lower DPPH IC_50_ and reducing‐power EC_50_ values, as expected for a soluble reference antioxidant. Accordingly, the present data do not demonstrate that PA‐AgNPs are superior to AE‐PAS or ascorbic acid as antioxidants. Nanoparticles can interfere with absorbance‐based assays through light scattering, adsorption, or intrinsic optical properties; therefore, the uncorrected PA‐AgNP DPPH value should be regarded as an apparent assay‐specific estimate (Kroll et al. 2012). Literature on phytochemical‐associated AgNP surfaces provides contextual support for altered reaction accessibility but does not establish enhanced stability or sustained activity in the present samples (Flieger et al. 2021; Anjali et al. 2025).

#### 3.4.2 Photoprotective Activity and Anti‐Inflammatory Activity

**FIGURE 4:**
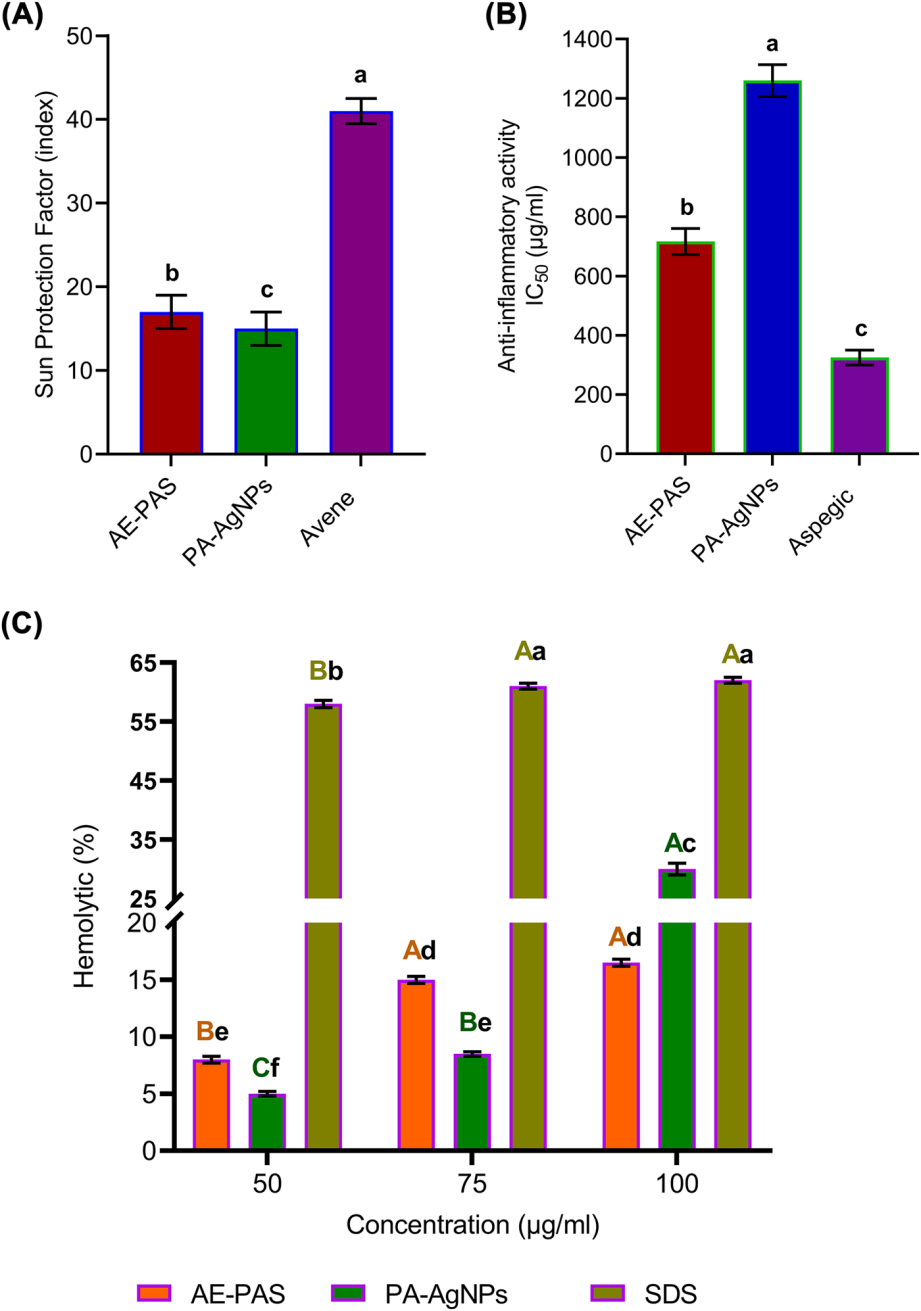
(**A**) The photoprotective activity or sun protection factor (SPF) of AE‐PAS, PA‐AgNPs, and commercial sunscreen (Avene), (**B**) anti‐inflammatory activity of AE‐PAS, PA‐AgNPs, and Aspegic (standard drug), and (**C**) hemolytic activity of AE‐PAS, PA‐AgNPs, and sodium dodecyl sulfate (SDS: positive control). Values are expressed as mean ± standard deviation (*n* = 3). Different uppercase letters indicate significant differences (*p* < 0.05) among concentrations of AE‐PAS and PA‐AgNPs, Aspegic, or SDS, while different lowercase letters indicate significant differences (*p* < 0.05) among concentration of AE‐PAS, PA‐AgNPs, and other agents as determined by one‐way ANOVA followed by post hoc analysis with Tukey's test. µg/mL, microgram/milliliters; IC_50_, half maximal inhibitory concentration. AE‐PAS, aqueous extract of *P. anisum* seeds.

The spectrophotometric SPF screening showed values of 17.05 ± 2.3 for AE‐PAS and 14.52 ± 1.8 for PA‐AgNPs, both lower than the commercial sunscreen Avene (40.64 ± 1.8; Figure 4A). The difference between AE‐PAS and PA‐AgNPs may reflect differences in UV absorbance, dispersion, light scattering, and accessibility of extract constituents. Natural polyphenols may contribute to UV absorption, whereas nanoparticle dispersion and light scattering may influence spectrophotometric SPF measurements. These contributions were not separated in the present assay (Salunkhe et al. 2022; Saewan and Jimtaisong 2015). These in vitro absorbance‐based SPF values constitute preliminary screening data and do not establish sunscreen efficacy, photostability, skin penetration, formulation safety, or suitability for topical application. Comparable absorbance‐based SPF screening has been reported for other plant‐mediated AgNP systems, but those studies do not validate topical performance of the present formulation (Laib et al. 2024; Mehwish et al. 2021). The anti‐inflammatory activity was assessed using the inhibition of egg albumin denaturation assay, a well‐established in vitro model that evaluates protein stabilization as an indicator of anti‐inflammatory potential (Figure 4B). AE‐PAS exhibited appreciable anti‐inflammatory activity with an IC_50_ of 721.79 ± 1.7 µg/mL. This observed activity represents in vitro protein denaturation inhibition, which serves as a simplified model of inflammation and does not directly reflect complex in vivo inflammatory pathways. Meanwhile, PA‐AgNPs showed a significantly higher IC_50_ value of 1261.38 ± 54 µg/mL (*p* < 0.05 vs. AE‐PAS), indicating lower potency in this specific assay. Although both samples were significantly less potent than the reference drug Aspegic (IC_50_ = 320.37 ± 25 µg/mL; *p* < 0.05), the differences in IC_50_ values should be interpreted within the limitations of the assay system rather than as definitive evidence of superior or inferior pharmacological efficacy.

Flavonoids and *P. anisum* preparations have been associated in previous studies with inflammation‐related responses (Al‐Khayri et al. 2022; Jawed et al. 2025). However, the present albumin‐denaturation assay did not measure COX or LOX activity, cytokines, prostaglandins, or oxidative‐stress biomarkers. The higher IC_50_ of PA‐AgNPs relative to AE‐PAS indicates lower apparent potency in this specific in vitro model and should not be reinterpreted as evidence of a beneficial nano‐biointerface mechanism. Particle–protein contact, surface reactivity, and phytochemical association may have influenced the assay response, but these explanations remain hypothetical. The present results therefore support only a preliminary protein‐denaturation‐inhibition response and do not establish COX inhibition, prostaglandin suppression, cytokine modulation, therapeutic anti‐inflammatory efficacy, or superiority to conventional NSAIDs. Related albumin‐denaturation or anti‐inflammatory screening has been reported for other plant‐mediated AgNP systems, but cross‐study comparisons remain limited by differences in the materials, concentrations, and test conditions (Laib et al. 2024; Chahardoli et al. 2025).

#### 3.4.3 Hemolytic Activity

Assessment of hemolytic activity is a fundamental step in determining the blood compatibility of bioactive extracts and nanomaterials, particularly for formulations with potential biomedical or therapeutic applications. In this study, the hemolytic effects of AE‐PAS and PA‐AgNPs were evaluated using human erythrocytes at concentrations of 50, 75, and 100 µg/mL. SDS was employed as a positive control to validate assay sensitivity (Figure 4C). As illustrated in Figure 4C, SDS induced extensive erythrocyte lysis at all tested concentrations, with hemolysis ranging from 57.76% to 61.64%, confirming that the positive control produced the expected high‐hemolysis response. In contrast, AE‐PAS exhibited a markedly lower hemolytic response. Hemolysis values increased modestly with concentration, reaching 8.35% ± 0.35%, 15.10% ± 0.39%, and 16.49% ± 0.34% at 50, 75, and 100 µg/mL, respectively. The concentration‐dependent increase was statistically significant (*p* < 0.05) yet remained substantially lower than the positive control. AE‐PAS produced concentration‐dependent hemolysis under the tested conditions. Because membrane‐active constituents were not specifically identified, quantified, or tested separately, the observed response cannot be assigned to saponins or to any other individual phytochemical.

PA‐AgNPs showed concentration‐dependent hemolysis of 5.20% ± 0.20%, 8.57% ± 0.58%, and 29.93% ± 1.63% at 50, 75, and 100 µg/mL, respectively. The values at 50 and 75 µg/mL were numerically lower than those of AE‐PAS but were reported as not significantly different at the corresponding concentrations (*p* > 0.05). At 100 µg/mL, PA‐AgNP hemolysis increased markedly and exceeded that of AE‐PAS (*p* < 0.05), indicating substantial concentration‐related erythrocyte damage. Hemolysis at 50 µg/mL was lower than that observed at the higher tested concentrations. However, this single in vitro result should not be classified as evidence of hemocompatibility or systemic safety because interpretation depends on the assay conditions, exposure duration, donor characteristics, and intended application (Saha et al. 2020). Potential explanations reported for silver‐nanoparticle‐associated hemolysis include particle–membrane interaction, released Ag^+^, and oxidative membrane damage (Do et al. 2025; Wypij et al. 2020; Andleeb et al. 2020). These processes were not measured in the present study, and the relative contributions of the particulate silver phase, released Ag^+^, and the phytochemical‐associated surface cannot be distinguished without appropriate controls and release measurements. Overall, PA‐AgNPs produced concentration‐dependent erythrocyte damage, particularly at 100 µg/mL. These in vitro findings do not establish suitability for systemic, topical, or localized use. Additional donor information, concentration‐response testing, nanoparticle‐specific controls, toxicological evaluation, and in vivo safety studies are required before route‐specific applications can be considered. Previous reports also emphasize that hemocompatibility and membrane responses vary with particle composition, surface properties, concentration, and exposure conditions (Laib et al. 2024; Andleeb et al. 2025; Saha et al. 2020).

#### 3.4.4 Antibacterial Activity

The antibacterial responses of AE‐PAS and PA‐AgNPs were screened against *E. coli*, *K. pneumoniae*, *P. aeruginosa*, *S. aureus*, and *B. subtilis* using agar well diffusion, with amoxicillin and cephalexin as reference controls (Table 2). PA‐AgNPs produced measurable inhibition zones against all tested strains, and larger zones were generally observed at higher tested concentrations. At 1000 µg/mL, inhibition zones were 18.5 ± 1.1 mm for *E. coli*, 18.0 ± 1.1 mm for *B. subtilis*, and 16.0 ± 1.1 mm for *S. aureus*. These observations indicate concentration‐associated agar‐diffusion responses under the tested conditions. They do not quantify inhibitory or bactericidal potency, and the relative contributions of the Ag/silver‐oxide phase, released Ag^+^, and the phytochemical‐associated surface cannot be determined from the present design (Fayed et al. 2024). AE‐PAS also produced measurable inhibition zones, ranging from 7.33 ± 0.57 to 9.33 ± 0.57 mm depending on concentration and strain. PA‐AgNPs produced larger zones than AE‐PAS in several comparisons; however, agar‐zone diameter is influenced by diffusion, solubility, particle aggregation, applied concentration, and material composition. Consequently, the difference in zone size cannot by itself demonstrate greater intrinsic antibacterial potency, reduced cytotoxicity, or biomedical suitability. The small strain panel also does not support a general conclusion that Gram‐negative or Gram‐positive organisms are preferentially susceptible. Agar‐ and well‐diffusion zone diameters are influenced by diffusion, solubility, inoculum, medium composition, and method conditions and therefore cannot substitute for MIC or MBC determination (Balouiri et al. 2016).

**TABLE 2:** Antibacterial activity of AE‐PAS and PA‐AgNPs against five pathogenic bacterial strains.

Samples	Concentration (µg/mL)	Zone inhibition (mm) (mean ± SD)				
		*Pseudomonas aeruginosa*	*Staphylococcus aureus*	*Bacillus subtilis*	*Klebsiella pneumoniae*	*Escherichia coli*
**PA‐AgNPs**	1000	14.5 ± 1.2^c^	16 ± 1.1^b^	18.00 ± 1.1^b^	14 ± 0.00^b^	18.5 ± 1.1^b^
	750	13 ± 1.1^d^	14.5 ± 1.1^d^	13.00 ± 1.1^c^	14 ± 1.1^b^	14.00 ± 0.5^c^
	500	12.5 ± 1.3^d^	12.5 ± 1.2^e^	12.00 ± 1.02^d^	13.7 ± 1.1^b^	12 ± 1.1^d^
	250	12 ± 1.2^e^	8.0 ± 0.6^f^	12 ± 1.1^d^	13.5 ± 1.1^b^	11.00 ± 0.7^e^
**AE‐PAS**	1000	9.33 ± 0.57^f^	8.00 ± 0.00^g^	8.67 ± 0.76^e^	8.33 ± 0.58^d^	8.0 0 ± 0.00^f^
	750	8.33 ± 0.29^g^	8.00 ± 0.00^g^	8.33 ± 0.29^ef^	8.33 ± 0.58^d^	7.73 ± 0.25^f^
	500	8.00 ± 0.00^g^	8.00 ± 0.00^g^	8.00 ± 0.00^ef^	8.00 ± 0.00^d^	7.41 ± 0.52^f^
	250	8.00 ± 0.00^g^	8.00 ± 0.00^g^	7.67 ± 0.29^f^	7.83 ± 0.29^d^	7.33 ± 0.57^f^
**Amoxicillin**	10	18 ± 0.05^b^	18 ± 0.03^a^	18 ± 0.056^b^	12 ± 0.02^c^	30 ± 0.02^a^
**Cephalexin**	30	20 ± 0.04^a^	15 ± 0.05^c^	19 ± 0.03^a^	17 ± 0.03^a^	18 ± 0.02^b^

*Note*: Values are presented as mean ± SD (*n* = 3). Different superscript letters within the same column indicate significant differences according to one‐way ANOVA followed by Tukey's HSD test at *p* < 0.05. Amoxicillin (10 µg/mL) and Cephalexin (30 µg/mL) were used as positive controls.

PA‐AgNPs at 1000 µg/mL produced an inhibition zone of 14.5 ± 1.2 mm against *P. aeruginosa*, compared with 20.0 ± 0.04 mm for cephalexin and 18.0 ± 0.05 mm for amoxicillin. Against *B. subtilis*, the PA‐AgNP zone was 18.0 ± 1.1 mm, compared with 18.0 ± 0.06 mm for amoxicillin and 19.0 ± 0.03 mm for cephalexin. Because the nanoparticles and antibiotics were tested at substantially different concentrations and possess different diffusion characteristics, these values should be treated only as descriptive agar‐diffusion benchmarks and not as evidence of equivalent potency. The higher nanoparticle concentration cannot be attributed to a different mechanism or interpreted as preserved intrinsic activity without MIC, MBC, and concentration‐normalized susceptibility data. Similar comparative agar‐diffusion observations have been reported for plant‐mediated silver nanoparticles, but they likewise do not establish concentration‐normalized equivalence to antibiotics (Adil et al. 2023; Fayed et al. 2024). Potential antibacterial mechanisms reported for silver‐based nanoparticles include membrane interaction, Ag^+^ release, oxidative stress, interference with enzymes, and effects of associated plant constituents (Prabhu and Poulose 2012; Girma et al. 2024; Laib et al. 2025). None of these mechanisms was directly measured in the present study. In addition, AgNO3 or another silver‐ion control and an appropriate material‐matched nanoparticle control were not included; therefore, the relative contributions of released Ag^+^, the particulate silver phase, and the phytochemical‐associated surface cannot be distinguished. The present findings should be described as preliminary agar well diffusion evidence only. MIC, MBC, time‐kill, release, and mechanistic studies are required before conclusions can be drawn regarding quantitative antibacterial potency, bactericidal activity, therapeutic applicability, or use as an alternative or adjunct to conventional antibiotics. These proposed mechanisms have also been discussed in earlier studies of plant‐derived and silver‐containing nanoparticle systems but were not experimentally distinguished here (Zayed et al. 2020; AlSalhi et al. 2016).

### 3.5 In Vivo Biological Activities of AE‐PAS and PA‐AgNPs

#### 3.5.1 Antidepressant‐Like Activity

**FIGURE 5:**
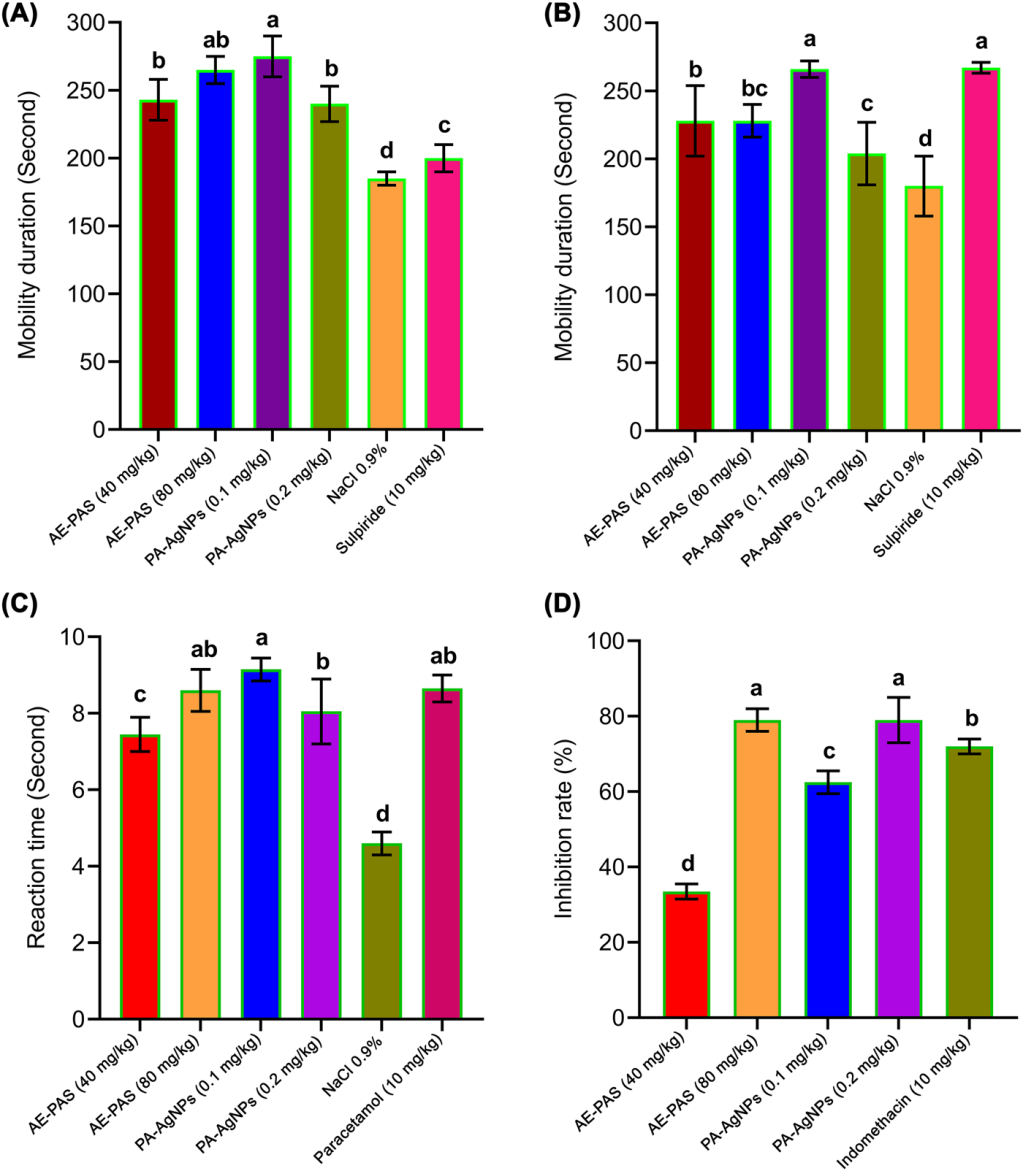
Behavioral effects of AE‐PAS and PA‐AgNPs in rats. (A) Forced‐swim and (B) tail‐suspension mobility after AE‐PAS, PA‐AgNPs, sulpiride (positive control), or 0.9% NaCl (negative control). (C) Hot‐plate reaction time after the same AE‐PAS and PA‐AgNP treatments, paracetamol (positive control), or 0.9% NaCl. (D) Inhibition of acetic‐acid‐induced writhing after the same AE‐PAS and PA‐AgNP treatments, indomethacin (positive control), or 0.9% NaCl. Values are mean ± SD (*n* = 5 animals/group). One‐way ANOVA with Tukey's HSD post hoc test; different superscript letters indicate *p* < 0.05. AE‐PAS, aqueous extract of *P. anisum* seeds.

The antidepressant‐like behavioral responses of AE‐PAS and PA‐AgNPs were evaluated using the FST (Figure 5A) and TST (Figure 5B). In the FST, mobility times were 242.62 ± 26.23 and 264.12 ± 7.36 s for AE‐PAS at 40 and 80 mg/kg, respectively; 274.72 ± 12.65 and 240 ± 13 s for PA‐AgNPs at 0.1 and 0.2 mg/kg, respectively; 199.34 ± 0.51 s for sulpiride; and 183.65 ± 2.68 s for saline. In the TST, mobility times were 227.50 ± 17.93 and 180.39 ± 8.13 s for AE‐PAS at 40 and 80 mg/kg, respectively; 266.50 ± 5.72 and 204.19 ± 23.18 s for PA‐AgNPs at 0.1 and 0.2 mg/kg, respectively; 267.33 ± 2.08 s for sulpiride; and 180.39 ± 17.93 s for saline. PA‐AgNPs at 0.1 mg/kg increased mobility in both assays, whereas responses at 0.2 mg/kg and following AE‐PAS treatment varied between models. The findings therefore do not demonstrate a monotonic dose‐response relationship, a hormetic response, or confirmed antidepressant efficacy. They indicate preliminary antidepressant‐like behavioral effects under the tested conditions. The larger behavioral responses observed for some PA‐AgNP groups at lower administered mass doses than AE‐PAS warrant further investigation, but the two formulations are not directly dose‐equivalent, and the present data do not establish superior potency. Blood–brain barrier penetration, central nervous system exposure, biodistribution, pharmacokinetics, MAO activity, dopamine or serotonin concentrations, receptor engagement, oxidative‐stress markers, and neuroinflammatory endpoints were not measured. Previous studies reported antidepressant‐like behavioral responses following *P. anisum* administration in mice, whereas studies of AgNP exposure indicate that central nervous system responses depend on particle characteristics, dose, and exposure conditions. Neither body of evidence establishes the mechanism of the present PA‐AgNP behavioral findings (Es‐safi et al. 2021; Dziendzikowska et al. 2021). The behavioral results should not be interpreted as evidence that PA‐AgNPs are an efficient or targeted treatment for depressive disorders. Hypotheses concerning nanoparticle‐associated delivery, phytochemical interactions, and neurobiological responses have been raised in related literature but require direct pharmacokinetic and mechanistic validation for PA‐AgNPs (Fayed et al. 2024; Do et al. 2025; Ghamarsoorat et al. 2025).

#### 3.5.2 Analgesic Activity

Analgesic‐related behavioral responses were evaluated using the hot‐plate and acetic‐acid‐induced writhing tests. In the hot‐plate test, reaction times were 7.51 ± 2.44 and 8.60 ± 0.90 s for AE‐PAS at 40 and 80 mg/kg, respectively; 9.18 ± 0.66 and 8.02 ± 0.87 s for PA‐AgNPs at 0.1 and 0.2 mg/kg, respectively; 8.62 ± 0.33 s for paracetamol; and 4.57 ± 0.37 s for saline (Figure 5C). In the writhing test, inhibition values were 33.33% ± 7.21% and 79.16% ± 14.43% for AE‐PAS at 40 and 80 mg/kg, respectively; 62.50% ± 12.5% and 79.16% ± 7.21% for PA‐AgNPs at 0.1 and 0.2 mg/kg, respectively; and 75.00% ± 12.5% for indomethacin (Figure 5D). These data show behavioral differences among the tested groups, but they do not demonstrate that nanoparticle formulation uniformly enhanced central or peripheral analgesic potency. The formulations were administered at different mass doses, and direct dose‐normalized equivalence was not established. The hot‐plate and acetic‐acid‐induced writhing tests provide different nociception‐related behavioral readouts, but neither test alone identifies the molecular mechanism responsible for the response (Deuis et al. 2017). Docking predictions for selected phytochemicals do not establish COX inhibition, prostaglandin suppression, or causal involvement in the behavioral findings (Pinzi and Rastelli 2019). Phenolic compounds have been investigated experimentally for COX inhibition in other systems, but those results cannot be transferred directly to the present formulation (Paulino et al. 2016). Pharmacokinetics, bioavailability, cellular uptake, blood–brain barrier penetration, neurotransmitters, inflammatory mediators, and tissue exposure were not measured. Therefore, the present findings indicate analgesic‐like behavioral responses under the tested conditions but do not establish superior analgesic efficacy, nanoparticle‐mediated potentiation, improved bioavailability, or a defined central or peripheral mechanism. Only one post‐administration observation interval was evaluated; future studies should assess multiple time points to characterize onset and duration. The exclusive use of male rats also limits generalizability, and appropriately designed studies including female animals are needed to assess possible sex‐dependent responses and safety. Broader literature has proposed links among nanoparticle‐associated redox, inflammatory, and neurobiological responses, but such pathways cannot be inferred from the present behavioral endpoints alone (Zhang et al. 2024).

### 3.6 Molecular Docking Study of Bioactive Compounds of AE‐PAS

**FIGURE 6:**
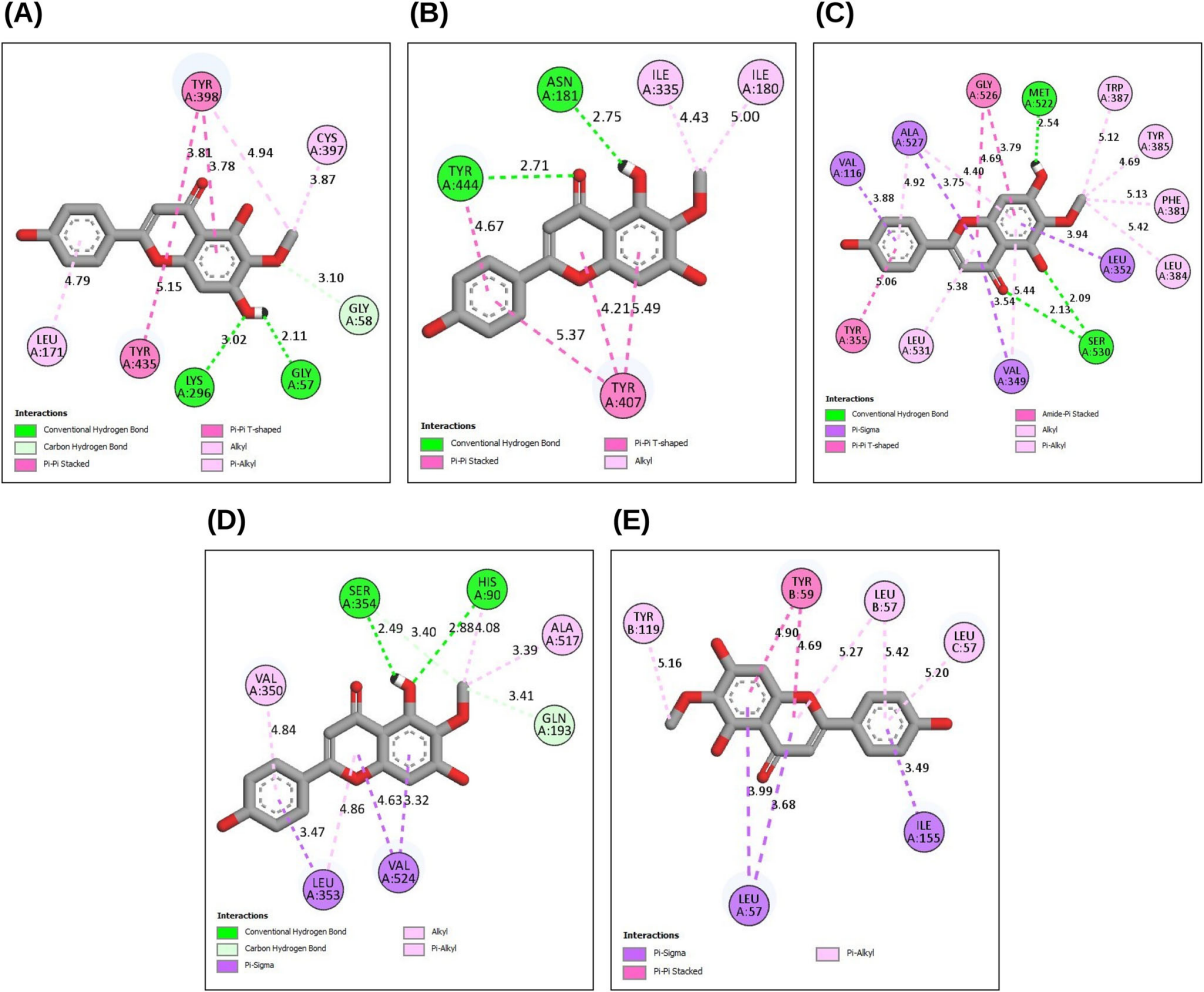
The visualization of resulting protein–ligand interactions in the two‐dimensional space of hispidulin with targeted receptors. (A) 1GOS, (B) 2Z5X, (C) 3KK6, (D) 4PH9, and (E) 9OJS.

**FIGURE 7:**
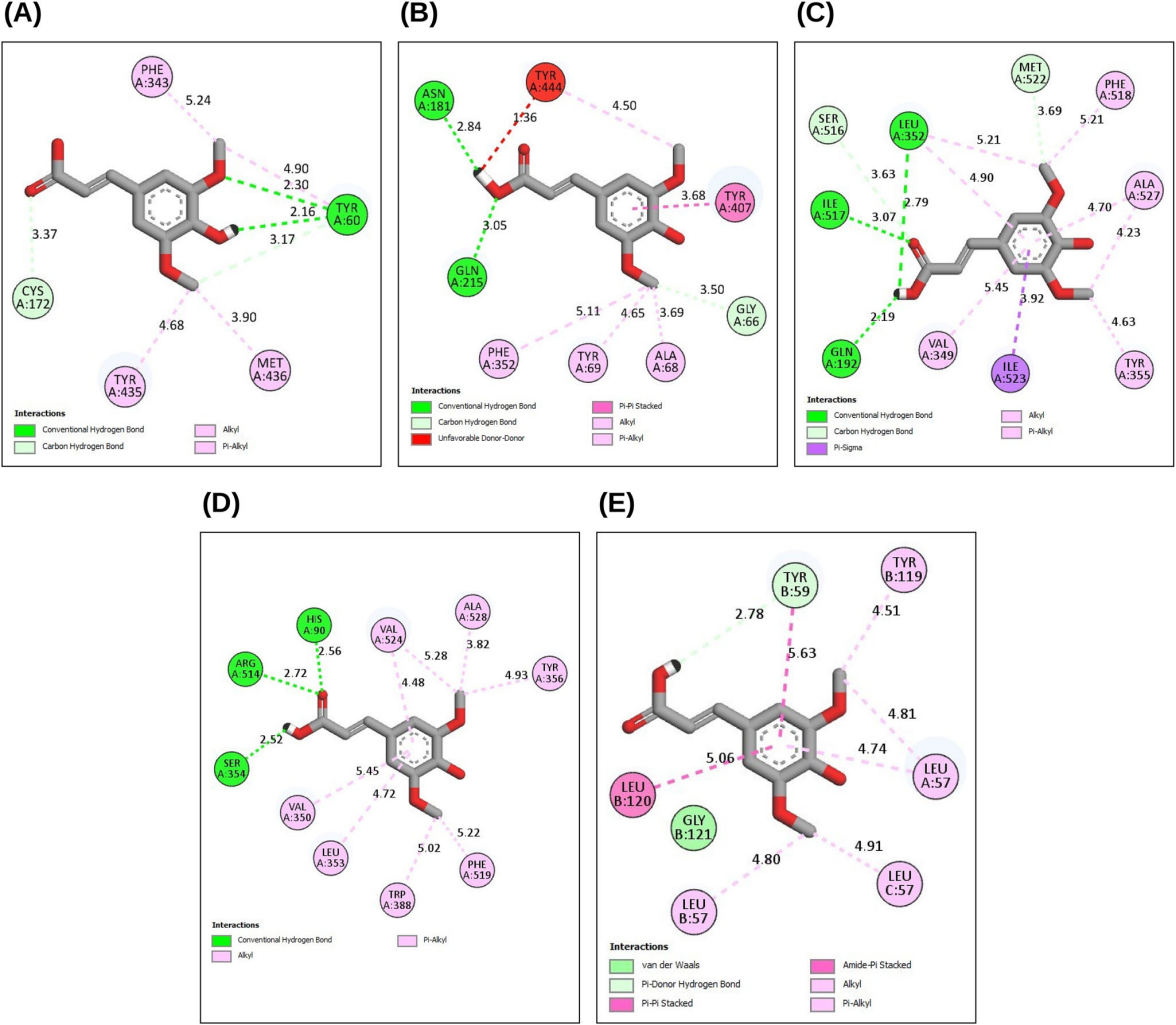
The visualization of resulting protein–ligand interactions in the two‐dimensional space of Sinapic acid with targeted receptors. (A) 1GOS, (B) 2Z5X, (C) 3KK6, (D) 4PH9, and (E) 9OJS.

**FIGURE 8:**
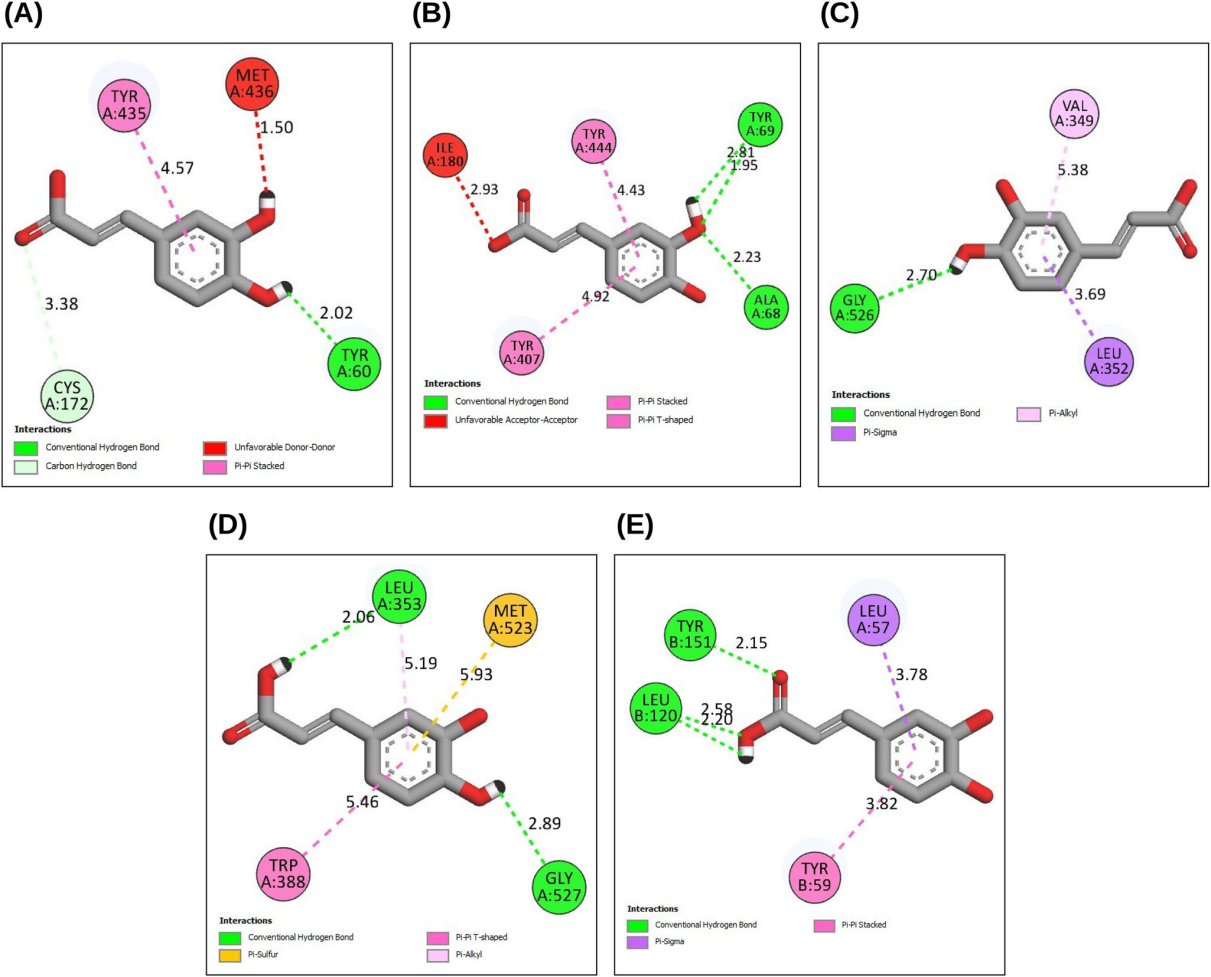
The visualization of resulting protein–ligand interactions in the two‐dimensional space of caffeic acid with targeted receptors. (A) 1GOS, (B) 2Z5X, (C) 3KK6, (D) 4PH9, and (E) 9OJS.

**FIGURE 9:**
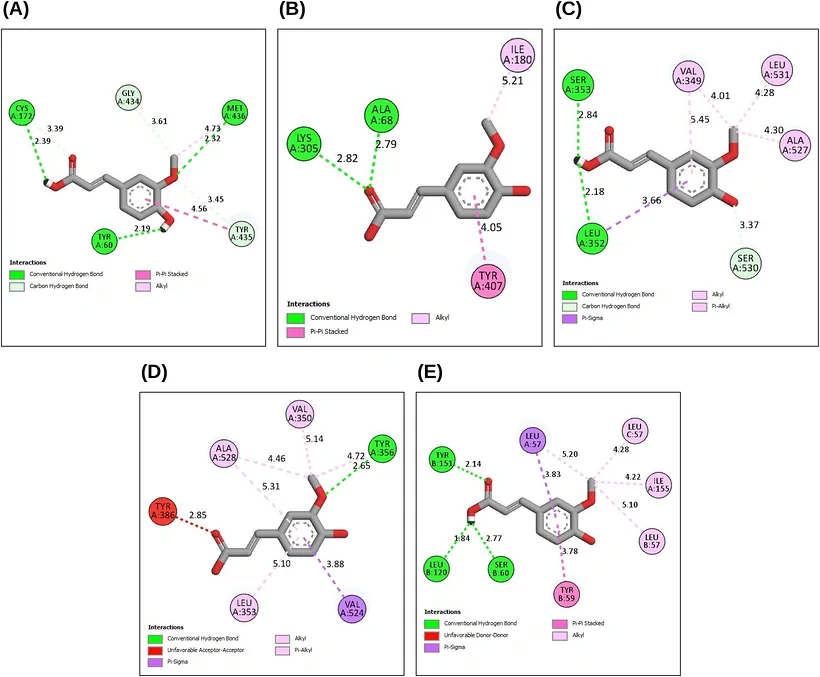
The visualization of resulting protein–ligand interactions in the two‐dimensional space of ferulic acid with targeted receptors. (A) 1GOS, (B) 2Z5X, (C) 3KK6, (D) 4PH9, and (E) 9OJS.

The in silico analysis generated hypothesis‐forming predictions concerning possible interactions between selected AE‐PAS phytochemicals and MAO‐A, MAO‐B, COX‐1, COX‐2, and TNF‐α structures. Re‐docking of native ligands yielded RMSD values of 0.31–1.56 Å (Table S2), supporting internal reproducibility of the selected docking protocol but not validating biological activity, target inhibition, or relevance to the intact nanoparticle formulation. Predicted binding scores for several flavonoids ranged from −8.0 to −9.6 kcal/mol, and some were comparable to or more favorable than scores obtained for the selected native ligands. Such score comparisons indicate favorable modeled poses under the chosen computational conditions; they do not establish simultaneous multi‐target modulation, therapeutic efficacy, or superiority to established ligands. The numerical scores and modeled contacts are presented in Table 3, Figures 6, 7, 8, 9, and Figure S2. Network pharmacology provides a conceptual basis for considering multi‐target actions, but favorable docking scores alone do not establish polypharmacology or biological activity (Hopkins 2008; Pinzi and Rastelli 2019).

**TABLE 3:** Predicted binding scores (kcal/mol) of selected phytochemicals with the target proteins.

	(Target proteins (PDB IDs))				
Compound	1GOS	2Z5X	3KK6	4PH9	9OJS
Hispidulin	−9.4	−9.2	−8.1	−8.3	−9.5
Sinapic acid	−6.9	−6.8	−6.9	−6.6	−6.9
Caffeic acid	−6.9	−7.5	−6.9	−6.7	−7.4
Ferulic acid	−7.1	−7.3	−7.1	−6.8	−7.6
Catechin	−9.4	−9.5	−7.8	−8.2	−9.1
Apigenin	−9.3	−9.4	−8.8	−8.4	−9.4
Luteolin	−9.6	−9.5	−8.5	−8.6	−9.4
Naringenin	−9.2	−9.4	−8.7	−8	−9.2
Resveratrol	−8.3	−8.6	−8.3	−7.9	−8.8

Several compounds produced favorable predicted scores against MAO‐A and MAO‐B, suggesting that biochemical testing of these phytochemicals may be warranted. However, MAO inhibition, neurotransmitter concentrations, and receptor‐level activity were not experimentally assessed; therefore, the docking results cannot explain the antidepressant‐like behavioral findings (Pinzi and Rastelli 2019). Moreover, favorable modeled poses against COX‐1, COX‐2, and TNF‐α do not demonstrate COX inhibition, prostaglandin suppression, TNF‐α modulation, anti‐inflammatory efficacy, or analgesic mechanism. These targets were selected because of their biological relevance, but computational predictions require enzyme, cellular, and in vivo validation. AE‐PAS contains multiple phytochemicals that may contribute independently or jointly to the measured responses. However, synergy among these constituents was not tested using combination designs, isobolographic analysis, fractionation, or target‐based experiments. Docking scores for individual free phytochemicals also cannot establish that the compounds retain the same availability or orientation when associated with the nanoparticle surface. Consequently, the present analysis does not demonstrate enhanced ligand–protein stability, optimized receptor engagement, reduced toxicity, broader therapeutic efficacy, or pharmacological superiority over single‐agent treatments. The docking findings should be regarded solely as hypotheses for future biochemical and mechanistic investigation (Chaachouay 2025; Efferth and Koch 2011).

## 4 Conclusion

This study provides evidence supporting the plant‐mediated formation of a silver/silver‐oxide‐containing material using aqueous *P. anisum* extract. UV–Vis, SEM, FTIR, and XRD indicated an SPR band at 421 nm, dry‐state SEM features of approximately 60–90 nm, an average crystallite‐domain size of 22.57 ± 1.2 nm, and associated organic functional groups. The in vitro assays showed redox‐related, albumin‐denaturation‐inhibitory, photoprotective‐screening, hemolytic, and agar‐diffusion responses, whereas the animal tests showed antidepressant‐like and analgesic‐related behavioral changes under the tested conditions. Molecular docking generated hypotheses regarding possible interactions of selected free phytochemicals with MAO‐A/B, COX‐1/2, and TNF‐α. These findings do not establish therapeutic efficacy, a molecular mechanism, improved bioavailability, or suitability for biomedical use. Major limitations include the absence of DLS, PDI, zeta potential, TEM, definitive phase analysis, Ag^+^‐release kinetics, long‐term stability data, silver‐ion and material‐matched nanoparticle controls, MIC/MBC and time‐kill testing, nanoparticle‐only blank correction in the DPPH assay, pharmacokinetic and biodistribution measurements, mechanistic biomarkers, broader toxicological testing, multiple post‐dose time points, and female‐animal evaluation. The pronounced hemolysis at 100 µg/mL further emphasizes the need for expanded safety assessment. Accordingly, all biological and docking results should be interpreted as preliminary, and comprehensive physicochemical, quantitative microbiological, pharmacokinetic, mechanistic, and safety studies are required before any biomedical translation is considered.

## Author Contributions

**Ibtissam Laib**: conceptualization, data curation, formal analysis, investigation, methodology, validation, visualization, supervision, writing – original draft, writing – review and editing. **Anis Ben Ali**: conceptualization, formal analysis, methodology, validation, visualization, writing – original draft. **Soundes Guedda**: data curation, investigation, methodology, validation. **Nardjes Guedda**: formal analysis, investigation, methodology, writing – review and editing. **Naglaa A. Abd‐Elkarim**: software, writing – original draft, writing – review and editing. **Yousef Benaissa**: conceptualization, data curation, writing – review and editing. **Nariman Essmat**: data curation, software, writing – review and editing. **Elhafnaoui Lanez**: formal analysis, investigation, writing – review and editing. **Mohamed F. Abouelenein**: data curation, software. **Ahmed I. Osman**: supervision, funding acquisition, resources, writing – review and editing. **Alaa Mahfouz**: writing – review and editing. **Ahmed K. Rashwan**: conceptualization, data curation, resources, software, investigation, visualization, writing – original draft, writing – review and editing.

## Funding

The authors have nothing to report.

## Ethics Statement

All procedures involving animal care and handling were conducted in accordance with ethical guidelines approved by the University of El Oued Ethics Committee (approval number: 04/S.C./FL/NS/EU/2025), and the study was registered under reference 4/2025.

## Conflicts of Interest

The authors declare no conflicts of interest.

## Supporting information




**Supporting Information**: jfds71388‐sup‐0001‐SuppMat.docx

## Data Availability

Data will be made available on request.
