# Supplementary material for: Biogenic Silver Nanoparticles Mediated by Phytoconstituents of the Edible Spice Pimpinella anisum: Characterization and In Vitro, In Vivo, and In Silico Biological Evaluation
Source: J Food Sci. 2026 Aug 31;91(9):e71388. doi: 10.1111/1750-3841.71388 (PMC13529928; doi:10.1111/1750-3841.71388)
Supplement: Supplementary file 1 [file JFDS-91-0-s001.docx]

**Biogenic silver nanoparticles mediated by phytoconstituents of the edible spice *Pimpinella anisum*: Characterization and *in vitro*, *in vivo*, and *in silico* biological evaluation**

Ibtissam Laib^1,2^, Anis Ben Ali^1,3^*, Soundes Guedda^1^, Nardjes Guedda^1^, Naglaa A. Abd-Elkarim^4^, Yousef Benaissa^5^, Nariman Essmat^6^, Elhafnaoui Lanez^1,7^, Mohamed F. Abouelenein^8^, Ahmed I. Osman^9^, Alaa Mahfouz^10^, Ahmed K. Rashwan^4,11^*

^1^ Department of Cellular and Molecular Biology, El Oued University, 39000, Algeria.

^2^ Laboratory of Biodiversity and Biotechnology Applications in Agriculture, University of El Oued, 39000, Algeria.

^3^ Laboratory of Biology, Environment and Health, Faculty of Natural and Life Sciences, University of El Oued, 39000, Algeria.

^4^ Department of Food and Dairy Sciences, Faculty of Agriculture, Qena University, Qena, 83523, Egypt.

^5^ VPRS Laboratory, Chemistry Department, Faculty of Mathematics and Matter Sciences. University of KASDI Merbah, Ouargla 30000, Algeria.

^6^ Department of Pharmacology and Toxicology, Faculty of Pharmacy, Zagazig University, Zagazig, 44519, Egypt.

^7^ Faculty of Exact Sciences, Department of Chemistry, VTRS Laboratory, University of El Oued, B.P.789, 39000 El Oued, Algeria.

^8^ Department of Insurance and Risk Management, College of Business, Imam Mohammad Ibn Saud Islamic University (IMSIU), Riyadh 11432, Riyadh, Saudi Arabia.

^9^ School of Sciences, Psychology, Arts and Humanities, Computing, Engineering & Sports, Canterbury Christ Church University, Canterbury CT1 1QU.

^10^ Medicinal and Aromatic Plants Department, Desert Research Center, 1 Mathaf El-Matariya St., El-Matariya, Cairo 11753, Egypt

^11^ Department of Food Science and Nutrition, College of Biosystems Engineering and Food Science, Zhejiang University, Hangzhou 310058, China.

* Corresponding authors:

**Dr. Ahmed I. Osman, Ph.D.**

School of Sciences, Psychology, Arts and Humanities, Computing, Engineering & Sports, Canterbury Christ Church University, Canterbury CT1 1QU.

E-mail: [ahmed.osman@canterbury.ac.uk](mailto:ahmed.osman@canterbury.ac.uk)

**Dr. Ahmed K. Rashwan, Ph.D.**

Department of Food and Dairy Sciences, Faculty of Agriculture, Qena University, Qena, 83523, Egypt.. E-mail: [a.rashwan@agr.svu.edu.eg](mailto:a.rashwan@agr.svu.edu.eg)

**Supplementary information**

| **Table S1**. The grid box centers and dimensions used of each protein with values unit of Å. | | | |
| --- | --- | --- | --- |
| **PDB ID** | **x-center (dimensions)** | **y-center (dimensions)** | **z-center (dimensions)** |
| 1GOS | 53.002 (50) | 156.279 (50) | 28.903 (50) |
| 2Z5X | 42.434 (50) | 25.987 (50) | -14.987 (50) |
| 3KK6 | -7.179 (50) | 44.127 (50) | -33.351 (50) |
| 4PH9 | 14.013 (50) | 25.041 (50) | 24.935 (50) |
| 9OJS | -13.905 (50) | -2.248 (50) | 20.009 (50) |
| Chain A was selected for each receptor | | | |

| Table S2. RMSD, resolution of targeted receptors, and binding energy values of re-docked native ligands. Superimposition of re-docked native ligand (pink) and native ligand (orange) | | | | | |
| --- | --- | --- | --- | --- | --- |
| PDB ID | **Resolution**  **(Å)** | **Native ligand** | | | **Native ligands superpose** |
|  |  | **BE** | **RMSD** | **Structure** |  |
| 1GOS | 3.00 | -5.7 | 1.5639 |  |  |
| 2Z5X | 2.20 | -8.3 | 1.2314 |  |  |
| 3KK6 | 2.75 | -10.6 | 0.9470 |  |  |
| 4PH9 | 1.81 | -7.7 | 0.3133 |  |  |
| 9OJS | 1.85 | -14.5 | 1.0098 |  |  |
| BE: Binding energy in kcal/mol, and RMSD: Root mean square distance in Å. | | | | | |

**Fig. S1.** LC-MS chromatographic profiles of *P. anisum* seed extract

**Fig. S2-1.** The visualization of resulting protein–ligand interactions in the two-dimensional space of catechin with targeted receptors

**Fig. S2-2.** The visualization of resulting protein–ligand interactions in the two-dimensional space of apigenin with targeted receptors

**Fig. S2-3.** The visualization of resulting protein–ligand interactions in the two-dimensional space of luteolin with targeted receptors

**Fig. S2-4.** The visualization of resulting protein–ligand interactions in the two-dimensional space of naringenin with targeted receptors

**Fig. S2-5.** The visualization of resulting protein–ligand interactions in the two-dimensional space of resveratrol with targeted receptors
